# Glucagon-like peptide-1 (GLP-1) and protective effects in cardiovascular disease: a new therapeutic approach for myocardial protection

**DOI:** 10.1186/1475-2840-12-90

**Published:** 2013-06-18

**Authors:** Ting C Zhao

**Affiliations:** 1 Cardiovascular Research laboratory, Department of Surgery, Roger Williams Medical Center, Boston University Medical School, 50 Maude Street, Providence, RI 02908, USA

**Keywords:** GLP-1, Insulin resistance, Heart, Cardiovascular disease, Diabetes

## Abstract

Glucagon-like peptide-1 (GLP-1) is a member of the proglucagon incretin family implicated in the control of appetite and satiety. GLP-1 has insulinotropic, insulinomimetic, and glucagonostatic effects, thereby exerting multiple complementary actions to lower blood glucose in subjects with type 2 diabetes mellitus. A major advantage over conventional insulin is the fact that the insulinotropic actions of GLP-1 are dependent upon ambient glucose concentration, mitigating the risks of hypoglycemia. Recently, the crucial role of GLP-1 in cardiovascular disease has been suggested in both preclinical and clinical studies. The experimental data indicate GLP-1 and its analogs to have direct effects on the cardiovascular system, in addition to their classic glucoregulatory actions. Clinically, beneficial effects of GLP-1 have also been demonstrated in patients with myocardial ischemia and heart failure. GLP-1 has recently been demonstrated to be a more effective alternative in treating myocardial injury. This paper provides a review on the current evidence supporting the use of GLP-1 in experimental animal models and human trials with the ischemic and non-ischemic heart and discusses their molecular mechanisms and potential as a new therapeutic approach.

## Introduction

Myocardial infarction contributes significantly to deaths related to coronary artery disease [1]. Diabetes mellitus threatens to become a global health crisis; treatment of diabetes and its implications constitutes a major health care expenditure. A significant proportion of diabetic patients are known to develop diabetic cardiomyopathy, with a high incidence of congestive heart failure. The continuous requirement for high-energy phosphates to perform mechanical work burdens the heart as metabolic requirements not shared by other organ system. Accordingly, substrate availability, oxidative phosphorylation, and high-energy phosphate transfer are critical to cardiac performance. While the heart is capable of utilizing a variety of available substrates to generate adenosine triphosphate, this metabolic flexibility is compromised under circumstances in which the heart is stressed, particularly by myocardial ischemia.

Diabetes causes suppressed glucose oxidation leading to inefficient energy production, enhanced fatty acid metabolism, and increased susceptibility to myocardial ischemia and reperfusion injury. In the ischemic myocardium, an increase in glucose uptake and subsequent ATP generated through glycolysis helps to sustain myocardial electric and mechanical performance, maintains cellular ultrastructure, promotes myocardial recovery. Accordingly, mechanism of enhancing myocardial energetic efficiency by stimulating glucose availability and utilization has led to the vigorous pursuit of therapeutic approaches designed to augment glucose uptake and oxidation. Although many therapeutic agents such as β-blockers and angiotensin-converting enzyme inhibitors are currently used to control cardiovascular diseases (CVD), there remains a substantially high incidence of CVD among diabetic patients, necessitating alternative strategies of targeted management [2]. One such area of interest is the ability to modulate myocardial glucose uptake and its impact on cardioprotection. Heart failure and myocardial infarction are insulin-resistant states that are associated with a significant risk for either concurrently having or subsequently developing newly-onset diabetes [3]. Insulin resistance is implicated in several potential adverse metabolic changes, including disturbances in insulin and glucose metabolism, which can affect energy supply and blood flow [4]. The injured myocardium develops an evolving dependence on glucose as its preferred metabolic substrate while development of myocardial insulin resistance is associated with the progression of heart failure and increased incidence as well as severity of the damaged hearts.

Insulin, glucose and potassium (GIK) are touted as useful metabolic adjuvant, associated with improvement of cardiac function in acute myocardial function, but the general acceptance of this therapeutic approach is limited by requirements for concomitant infusion of glucose and concerns regarding hypoglycemia. Glucagon-like peptide-1 (GLP-1) is a naturally occurring incretin that is implicated in the control of appetite and satiety. GLP-1 has been studied extensively in type 2 diabetes as a novel insulinotropic peptide whose actions are predicated upon the ambient glucose concentration. The experimental studies and clinical data demonstrated that utilizing GLP-1 as a treatment in patients with heart failure improved cardiac function. We and others further have shown that GLP-1 protected the heart against ischemic injury. In this brief review, we will summarize recent progress about the promising role of GLP-1 in myocardial protection and signaling pathway.

### GLP-1 and its biological role

Glucagon-like peptide-1 (GLP-1) is a member of the pro-glucagon incretin family implicated in the control of appetite and satiety [5]. GLP-1 acts through GLP-1 receptor (GLP-1R), a 463 amino-acid member of the G protein-coupled receptor (GPCR) superfamily [6]. Bioactive GLP-1 exists in two equipotent molecular forms: GLP-1^7–37^ and GLP-1^7–36 amide^. GLP-1 is rapidly cleaved by DP IV, which results in the generation of largely inactive molecular GLP-1^9-36 amide^ and GLP-1^7-37^ forms. The majority of GLP-1 leaving the intestinal venous circulation has already been cleaved by DP IV expressed in capillary surrounding gut L cells, which provides an estimated half-life of 1–2 minutes for intact GLP-1 in vivo [7]. The GLP-1 receptor is widely distributed in tissues, including brain, pancreas, intestine, lung, stomach, and kidney. The effects of GLP-1 appear to be both insulinotropic and insulinomimetic, depending on the ambient glucose concentration. GLP-1 is studied extensively in type 2 diabetes as a novel insulinotropic peptide whose actions are predicated upon the ambient glucose concentration. The actions of GLP-1 to stimulate pancreatic insulin release are attenuated at a glucose concentration less than 4 mmolar. In addition, GLP-1 also exerts actions independent of insulin secretion, such as inhibiting glucagon secretion, gastric emptying, and gastric acid secretion while reducing food intake after both intracerebroventricular and peripheral administration [8]. There is accumulated evidence showing that administration of GLP-1 agonists promotes differentiation of functional β cells both in vitro and in vivo [9,10]. Furthermore, administration of extendin-4 in the neonatal period to rats following induction of experimental intrauterine growth retardation is associated with a reduced incidence of diabetes due to increased β-cell mass cell proliferation [11]. Mechanisms of GLP-1 in the regulation of β-cell mass remain unclear, but may involve MAP kinase, PKCζ, and phosphatidylinositol-3 kinase.

In addition, evidence suggests that GLP-1 acting outside of the pancreas is also important for regulation of glucose metabolism. GLP-1 also has been shown to stimulate glucose disposal via an insulin-independent mechanism(s) [12]. Myocardial receptors for GLP-1 are identified in rodents and human myocardium [13,14]. Although receptors for GLP-1 are found in a variety of tissues including the heart, current evidence exists mainly to support the role of GLP as a modulator of pancreatic hormone release [15]. GLP-1 has been administered as a continuous infusion in type 2 diabetics with impressive insulin-sensitizing effects, reduced insulin resistance in skeletal muscle and adipose tissue, and improvements in insulin-mediated glucose uptake.

### Insulin resistance in myocardium

Insulin resistance is commonly defined as a decreased response of glucose uptake to the stimulatory effect of insulin [16]. Glucose is an especially important substrate for the heart [17], and influences both normal cardiac function and the response of the heart to ischemia that promotes glucose uptake and decreases the utilization of free fatty acids by the human heart [18]. A decrease in glycolysis has been shown in various animal models of heart failure, and heart failure is linked with insulin resistance. On the other hand, patients with chronic heart failure due to coronary artery disease are more likely to have abnormalities in glucose metabolism than are patients with congestive heart failure (CHF) due to idiopathic dilated cardiomyopathy [19]. It has been demonstrated that in patients with myocardial infarction, metabolic syndrome and diabetes were highly prevalent and associated with higher risks of deaths and major cardiovascular events [20]. In addition, euglycaemic hyperinsulinaemic clamp techniques reveal that insulin-stimulated glucose uptakes was 20% lower in congestive heart failure patients versus healthy subjects. This is further extended by clinical finding showing that CHF is associated with marked insulin resistance, characterized by both fasting and stimulated hyperinsulinemia. Using laboratory experimental rat myocardial infarction, Clarke’s group has demonstrated that insulin-stimulated D[2-^3^H] glucose uptake was 42% lower in isolated perfused infarcted hearts, was and this was accompanied by a decrease in expression of glucose transporter (GLUT-4), and negatively correlated with ejection fraction and with fasting plasma FFA concentration [21]. Notably, low glucose uptake in chronic myocardial infarction was also directly in line with a three-fold faster ATP hydrolysis rate, measured using phosphorus-31 magnetic resonance spectroscopy.

### Experimental studies of GLP-1 in myocardial protection

A plethora of experimental data has been generated concerning a role of GLP-1 in diabetes, but very limited evidence has focused on its cardiovascular effect. Both GLP-1(7-36) amide and the GLP-1 receptor agonist, exendin-4 are shown to increase heart rate and blood pressure in both anesthetized and conscious restrained rats, although the mechanisms are controversial [22,23]. In vitro studies have failed to show any effects of GLP-1 on cardiac myocyte contractility. Very recently, promising clinical data from Shannon’s lab showed that GLP-1 infusion improved regional and global function in patients with acute myocardial ischemia and severe systolic dysfunction after successful primary angioplasty [24]. Furthermore, in pacing-induced heart failure model, the stimulation of GLP signaling with GLP-1 has also been demonstrated to improve cardiac performance in conscious dogs with dilated cardiomyopathy [25]. Infusion of GLP-1 was associated with a marked improvement in LV systolic function and diastolic function in decompensated heart failure. By using established isovolumetric isolated perfused rat hearts, we observed that activation of GLP-1 receptor with GLP-1 significantly mitigated myocardial injury as indicated by an improvement in recovery of developed pressure and end-diastolic pressure as well as rate pressure products [26]. Moreover, post-ischemic myocardial improvement was accompanied by a significant reduction in myocardial necrosis. GLP-1 caused an increase in myocardial glucose uptake in isolated hearts, which was in agreement with a previous study in which the GLP-1 promoted myocardial glucose uptake in conscious dogs with pacing induced dilated cardiomyopathy [25]. This may reflect a fact that the protective effect of GLP-1 on ischemic heart was attributed to overcoming insulin resistance under ischemic conditions. In agreement with our studies, evidence from Yellon’s observations suggests that GLP-1 added before ischemia induced a significant reduction of infarct size as compared to the control group. However, this protection was abrogated by administration of GLP-1 antagonist exendin (9-39) and inhibition of PI3 kinase [27]. However, Kavianpour et al. reported that GLP-1 (7-36) was not found to alter myocardial glucose utilization; hemodynamic variables and consequent infarction changed in porcin myocardium despite GLP-1 increased insulin secretion and decreased blood uptake [28]. The discrepancies of GLP-1 in these studies definitely depend on the model (in vivo/in vitro), species, and the duration of GLP-1 (short/sustained half life). Native GLP-1 is rapidly degraded by dipeptidyl peptidase-IV (DPP-IV) in the blood stream. Accordingly, the direct cardiovascular response of GLP-1 may be masked by the effective fragments under in vivo conditions [29].

### Clinical studies of GLP-1 in patients

The promising data obtained in experimental studies suggest that the therapeutic strategies based on incretins may preserve cardiomyocyte viability, increase metabolic efficiency, and inhibit the structural and functional remodeling that occurs in the ischemic and the failing heart. Against the backdrop of an expansive body of evidence indicating salutary cardiovascular effects of GLP-1 in the experimental animal model, there have been several phase 2 trials of GLP-1 in humans with cardiovascular diseases [5,24,30]. Shannon’s group is the first to demonstrate that infusion of GLP-1(7-36) (1.5 pmol/kg/min) for 72 hr in patients with left ventricular dysfunction after MI improved global and regional left ventricular wall motion scores and reduced hospital stay and in-hospital mortality. These effects remain detectable even several weeks after hospital discharge [5]. The benefits of GLP-1 on LVEF were evident in both diabetic and non-diabetic patients, as well as in patients with anterior (left anterior descending artery) and non-anterior AMI. Sokos et al. reported that a long-term infusion of GLP-1 improves both LVEF and functional capacity in human patients with advanced heart failure [30]. In a single-center pilot study, 20 obese patients with LVEF≤40% and NYHA class III or IV heart failure were divided to receive a continuous subcutaneous infusion of GLP-1 or a small volume of saline as a control over 5 weeks. All patients in the study were on a standard, stable heart failure medication regimen. The group treated with GLP-1 had significantly improved LVEF, maximum myocardial ventilation oxygen consumption, 6-minute walk distance and Minnesota Living with Heart failure Quality of Life scores. GLP-1 also led to improved glycemic control with an increase in plasma insulin and decrease in plasma NEFA levels. The promising results from the above clinical studies were further corroborated with additional recent data obtained in a pilot study performed in 20 patients after percutaneous coronary intervention confirming the cardioprotective effects of GLP-1, ameliorating left ventricular dysfunction after the ischemic event [31]. Another pilot study showed that treatment with GLP-1 (7-36) (1.2 pmol/kg/min) improved left ventricular function in response to stress in 14 patients with coronary artery disease (CAD) [32]. This observation was furthered supported with an additional report that 172 patients treated with exenatide (0.12 μg/min) for 6 hr after ST-Segment elevation MI exhibited a significant increase in myocardial salvage when compared with the placebo group.

### Anti-hypertensive effect of GLP-1

The cardioprotective effects of GLP-1 have also been reported in the in vivo hypertension animal models in which GLP-1 significantly improved the survival rates of the Spontaneously Hypertensive, Heart Failure–prone (SHHF) rats that demonstrated preserved LV function and LV mass index [33]. Such anti-hypertentive action has been demonstrated in Dahl salt-sensitive (DSS) rats in which continuous administration of recombinant GLP-1 attenuated the development of hypertension [34]. The prevention of hypertension by GLP-1 was directly linked to decreased LV hypotrophy in DSS as well as higher levels of urine flow and sodium excretion, contributing to the comprehensive anti-hypertension effects of GLP-1. Liu Q et al. reported that treatment of AC3174, an exenatide-related GLP-1R agonist, antagonized the hypertensive effect and prevented the development of cardiac hypertrophy in DSS model [35]. These data were further confirmed by the observations that inhibition of DPP-4 through the administration of Sitagliptin to increase the level of biologically active intact GLP-1 significantly attenuated high blood pressure in young pre-hypertensive spontaneously hypertensive rats (SHRs) [36]. Additionally, function and expression of Na/H exchanger isoform 3 (NHE3) decreased but there was increased urine flow and sodium excretion in the tested young SHRs. Pooled data from six trials showed that 6 months of exenatide treatment significantly reduced systolic blood pressure in patients with type 2 diabetes [37]. Liraglutide was evaluated in a series of phase 3 trials and it demonstrated a promising anti-systolic blood pressure effect in combination with other anti-diabetes drugs like metformin [38]. Using transgenic mouse model, it has recently been demonstrated that that cardiac GLP-1R activation promotes the secretion of atrial natriuretic peptide (ANP) and a reduction of blood pressure. LGLP-1R agonist liraglutide did not result in ANP secretion, vasorelaxation, or lower blood pressure in Glp1rdeficient mice, revealing a gut-heart GLP-1R-dependent and ANP-dependent axis that regulates blood pressure [39].

### GLP-1 and anti-obese effects

The last few decades have witnessed a global rise in adult obesity of epidemic proportions. The potential impact of this is emphasized when one considers that body mass index (BMI) is a powerful predictor of death, type 2 diabetes (T2DM), and cardiovascular morbidity and mortality [40]. Weight gain and obesity are associated with structural and functional changes of the cardiovascular system including left atrial and ventricular remodeling, hemodynamic alterations, autonomic dysfunction, and diastolic dysfunction. Moreover, diabetic cardiomyopathy is characterized by an adverse structural and functional cardiac phenotype [41]. For persons with diabetes or obesity, the chief goal is to avoid the common cardiovascular sequelae. In past years, numerous drugs have been approved for treatment of obesity. However, most of them are withdrawn from the market because of their adverse effects. Li C et al. observed that Addition of liraglutide to abdominally obese, insulin-treated patients led to improvement in glycemic control similar to that achieved by increasing insulin dosage [42]. Adding liraglutide to insulin also induced a significant reduction in body weight and waist circumference, suggesting that Liraglutide may be the best treatment option for poorly controlled type 2 Diabetes and abdominal obesity. In agreement with this evidence, Fujishima et al., demonstrated that Liraglutide improved visceral fat adiposity, produced meaningful weight loss, and significantly improved eating behavior in obese Japanese patients with type 2 diabetes. The effect of GLP-1 receptor agonist treatment on endothelial function has not been well-described in humans [43,44]. Kelly et al. demonstrated that three months of therapy through GLP-1 agonists (exenatide vs metformin) exerted similar effects on microvascular endothelial function, inflammation, oxidative stress, and vascular activation in patients with obesity and pre-diabetes [45].

### Signaling pathway involved by GLP-1 in myocardial protection

We observed abundant expression of GLP-1 receptors in the rat myocardium. However, the mechanisms whereby GLP-1 receptors couple to intracellular effectors in extrapancretic tissues, such as the heart, remain largely unexplored. In turn, we observed that GLP-1 and insulin had comparable effects on myocardial glucose uptake, but via different cellular mechanisms. We have found that p38 mitogen-activated protein kinase serves as an important mechanism through which GLP-1 modulates myocardial injury. In addition, the co-ordination between PI3K and nitric oxides in modulating cardiac function has recently been established.

### The mitogen-activated protein kinases (MAPKs)

MAPKs play a central role in the transmission of signals from cell surface receptors and various environmental cues to the transcriptional machinery in the nucleus involved in cell growth, differentiation, and transformation [46,47]. Several distinct MAPK subfamilies have been characterized in cardiac tissue, including the p38 MAP kinase, the stressed-activated protein kinase/c-Jun N-terminal kinase (JUN kinase), the extracellular-responsive kinase (ERKS), big MAPK-1(ERK5) [48]. p38 can be activated by various stresses including cytokines and I/R [49]. p38 has been shown to be protective in several models [50]. The p38 family of mitogen-activated protein kinases has been shown to play an important role in mediating stress-induced signaling in mammalian cells. There are two predominant isoforms of p38 in heart, α and β. Overexpression of p38 β has been shown to induce hypertrophic responses and to promote survival of myocytes, whereas activation of p38 α antagonizes these effects and leads to cell death [51].

Our previous studies showed that activation of p38 protected the heart against I/R injury [52]. In line with our observation, activation of p38 with preconditioning stimuli or over-expression of MKK3/MKK6 has been reported to protect the heart against myocardial I/R injury [53-58]. Mutation of p38 β isoform also resulted in increased myocardial injury [59]. However, during lethal ischemia, ischemia and reperfusion as well as post-conditioning p38 inhibition has been shown to result in protection [60,61]. Transgenic mice expressing a cardiac dominant-negative (Dn) p38 α antagonized cardiac I/R injury, and disruption of a single copy of p38 α in mice was reported to be less susceptible to myocardial I/R injury [62,63]. Dominant negative over-expression of p38 α transgenic mice show enhanced cardiac hypertrophy following aortic banding [64]. Dn-p38α mice had a markedly reduced infarct size and increased ventricular systolic function flowing chronic infarction [65]. These conflicts remain unclear. These discrepancies may be explained by strength of p38 activation and activation of different isoforms, vastly different stress conditions, and animal species [66-68]. During low flow ischemia, we documented that both GLP-1 and insulin-mediated glucose uptake did not involve Akt-1 activation in the post-ischemic myocardium [26]. It is intriguing to reveal from our recent observation that GLP-1 was associated with increased p38 activities. However, based on our current studies, it remains unknown if these unique signaling components are only epiphenomenon or crucial for GLP-1 to play a protective effect. If so, what p38 isoform/nitric oxide synthase would be dominant in regulation of cardioprotection afforded by GLP-1.

### PI3K and nitric oxides

The importance of p38/nitric oxide has been addressed based on our recent findings [52]. Inhibitors of PI3K and p42/44 mitogen-activated protein kinase (LY294002 and UO126), respectively as well as of p70S6K rapamycin abolished the GLP-1-induced infarct size limitation in rat hearts, suggesting that PI3K and p42/44 are involved in the myocardial protection elicited by GLP-1 [27,69]. These results indicate GLP-mediated activation of reperfusion injury salvages kinase pathway, reminiscent of the GLP-1 coupling to PI3K-akt-signlaing in insulin-producing cells [70]. In addition, insulin mediated glucose uptake was associated with Akt-1 phosphorylation and GLUT 4 translocation [26]. In contrast, GLP-1 did not increase Akt-1 phosphorylation and GLUT 4 translocation, but did result in increased GLUT 1 expression in the sarcolemma. Administration of L-N^G^-Nitroarginine Methyl Ester (L-NAME) attenuated this protective effect in mouse model [71], pointing to GLP-1-mediated cardioprotection through modulation of nitric oxides. Some studies also reveal that GLP-1 has a dose-dependent vasorelaxant effect, which is mediated by both endothelium and nitric oxide [71,72]. Golpon et al. showed that NG-nitro-Larginine methyl ester treatment inhibits endothelial NOS (eNOS) abolished GLP-1 mediated vasorelaxation of rat pulmonary arteries [72]. In addition, other studies suggested that the vasorelaxant effects of GLP-1 were mediated by activation of AMP or KATP channels independently of nitric oxide and the endothelium [73,74]. GLP-1-activated pathway uses cyclic adenosine monophosphate (cAMP) in insulin producing cells [70], and a cAMP-dependent pathway would be essential for the pro-survival action of GLP-1 in the heart [27]. GLP-1-mediated stimulation of cAMP in cardiac myocytes has been demonstrated [13].

### Other signaling pathways

The endoplasmic reticulum (ER) is a multifunctional organelle responsible for the synthesis and folding of proteins as well as calcium storage and signaling. Perturbations of ER function cause ER stress leading to the unfolded protein response, which includes inhibition of protein synthesis, protein refolding, and clearance of misfolded proteins [75]. Growing evidence links the ER to pathologies such as diabetes mellitus, obesity, liver, heart, renal and neurodegenerative diseases, endothelial dysfunction, atherosclerosis, and cancer [76]. Recent discoveries regarding the role of inflammation, mitochondrial dysfunction, and ER stress in obesity have advanced our understanding of how insulin resistance develops in peripheral organs [77]. A recent observation using a diabetic cardiomyopathy rat model reveals that the GLP-1 analog liraglutide improved cardiac function, which is accompanied with a decrease in activating transcription factor 4 (ATF4) and TNF receptor associated factor 2 (TRAF2) and the down-regulation of Grp78 and caspase-3. This suggests that GLP-1-induced cardioprotection may be related to the inactivation of the ER stress signaling pathway [78].

## Conclusion

**Figure 1:**
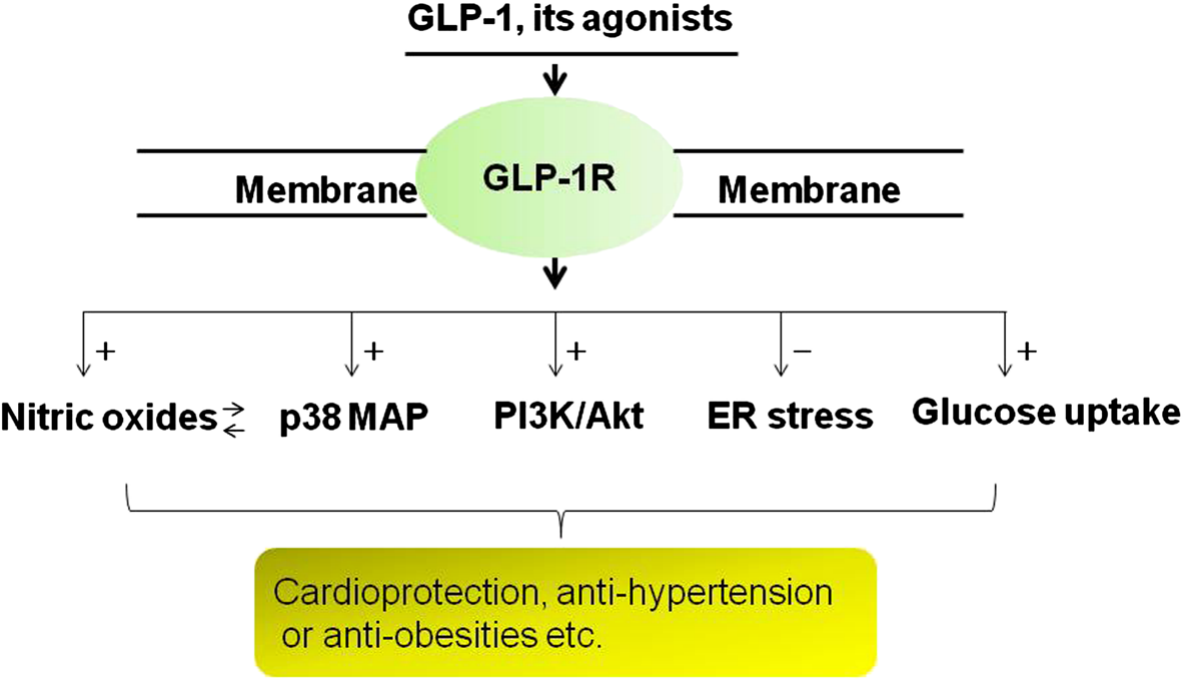
**Proposed signaling pathways induced by GLP-1.** GLP-1: glucagon-like peptide-1; GLP-1R: glucagon-like peptide-1 receptor; p38 MAPK: p38 mitogen-activated protein kinase; PI3K: phosphatidylinositide 3-kinase; ER: endoplasmic reticulum.

GLP-1 recently attracted attention as a therapeutic strategy for diabetes, heart diseases, and obesity. Metabolic modulation of post-ischemic myocardium and advanced left ventricular dysfunction is an important and emerging area of therapeutic investigation. Conventional approaches using insulin have been proven ineffective, development of new strategies to promote glucose uptake is a promising initiative. We and others have established the significance of GLP-1 in protect the heart against acute myocardial ischemic injury. Recently, a significant number of studies have indicated beneficial effects of GLP-1 on cardiovascular function, which appears to justify the usage of GLP-1 in the treatment of cardiovascular diseases. Exploration of its downstream signaling pathway using integrative molecular and cellular approaches and evaluation of its clinical outcome will provide direct evidence to support potential clinical implication (summarized in Figure 1).

## Competing interests

The author declares that he has no competing interest.
